# Marine viruses discovered via metagenomics shed light on viral strategies throughout the oceans

**DOI:** 10.1038/ncomms15955

**Published:** 2017-07-05

**Authors:** Felipe H. Coutinho, Cynthia B. Silveira, Gustavo B. Gregoracci, Cristiane C. Thompson, Robert A. Edwards, Corina P. D. Brussaard, Bas E. Dutilh, Fabiano L. Thompson

**Affiliations:** 1 Instituto de Biologia (IB), Universidade Federal do Rio de Janeiro (UFRJ), Rio de Janeiro 21944970, Brazil; 2 Centre for Molecular and Biomolecular Informatics (CMBI), Radboud Institute for Molecular Life Sciences, Radboud University Medical Centre, Nijmegen 6500 HB, The Netherlands; 3 Theoretical Biology and Bioinformatics, Utrecht University (UU), Utrecht 3584 CH, The Netherlands; 4 Biology Department, San Diego State University (SDSU), San Diego, California 92182, USA; 5 Departamento de Ciências do Mar, Universidade Federal de São Paulo (UNIFESP), Baixada Santista 11070100, Brazil; 6 Department of Marine Microbiology and Biogeochemistry, NIOZ Royal Netherlands Institute for Sea Research, and University of Utrecht, PO Box 59, 1790 AB Den Burg Texel, The Netherlands; 7 Department of Aquatic Microbiology, Institute for Biodiversity and Ecosystem Dynamics (IBED), University of Amsterdam, Amsterdam 1090 GE, The Netherlands; 8 Universidade Federal do Rio de Janeiro (UFRJ)/COPPE/SAGE, Rio de Janeiro 21941950, Brazil

## Abstract

Marine viruses are key drivers of host diversity, population dynamics and biogeochemical cycling and contribute to the daily flux of billions of tons of organic matter. Despite recent advancements in metagenomics, much of their biodiversity remains uncharacterized. Here we report a data set of 27,346 marine virome contigs that includes 44 complete genomes. These outnumber all currently known phage genomes in marine habitats and include members of previously uncharacterized lineages. We designed a new method for host prediction based on co-occurrence associations that reveals these viruses infect dominant members of the marine microbiome such as *Prochlorococcus* and *Pelagibacter*. A negative association between host abundance and the virus-to-host ratio supports the recently proposed Piggyback-the-Winner model of reduced phage lysis at higher host densities. An analysis of the abundance patterns of viruses throughout the oceans revealed how marine viral communities adapt to various seasonal, temperature and photic regimes according to targeted hosts and the diversity of auxiliary metabolic genes.

## Abstract (web summary)

The understanding of marine virus diversity and function is in its infancy. Here, Coutinho *et al*. assemble a data set of new viral contigs and use co-occurrence analyses to identify the putative hosts, elucidate infection strategies and viral strategies for exploiting their hosts.

Marine viruses regulate the community composition of their microbial hosts by selectively killing them. Viral lysis mediates the transfer of organic matter between live biomass and the dissolved organic carbon pool through the viral shunt^1^,^2^. The release of organic matter via the viral shunt is estimated to be close to 10 billion tons of carbon per day and is considered a fundamental step in nutrient cycling that fuels the productivity of the oceans^2^,^3^,^4^,^5^. Associations between the viral and host abundance have been described by the Kill-the-Winner theory that postulates that the higher the growth rate of a microorganism, the more likely it is to be targeted by a lytic viral infection^2^,^6^,^7^,^8^,^9^. This trait allows the slow-growing prokaryotes to reach a higher abundance than the fast growers because they are subject to fewer lytic infections^8^,^10^. The discovery that the decrease in the virus-to-microbe ratio at a high host abundance that is not associated with host resistance to infections has expanded this model^11^,^12^: the recently proposed Piggyback-the-Winner theory of virus–host interactions postulates that at a high host abundance, viruses favour lysogenic infections and integrate into the host genome when those are thriving instead of killing them through a lytic cycle^11^,^13^. The influence of viruses on the marine microbial community is not limited to killing. Viruses that infect bacteria and archaea, known as phages, can mediate genetic transduction. Host organisms can acquire viral genetic material via this mechanism and vice versa. Such an exchange of DNA may potentially result in new functional genes that are advantageous to the fitness of the virus or add to the diversification of the host metabolism^2^,^14^,^15^. Moreover, viruses may encode auxiliary metabolic genes that can be expressed during infection to steer central pathways of host metabolism such as photosynthesis and nutrient acquisition towards processes that favour the production of new viral particles^2^,^14^,^15^,^16^,^17^,^18^.

Metagenomics has become a powerful tool to characterize the biological diversity of viral communities *in situ*, but these studies often rely on reference databases for read annotation. The lack of a comprehensive database of marine viral genomes leads to poor virome (viral metagenome) read annotation^19^,^20^,^21^,^22^,^23^. Consequently, any taxonomic or functional analysis of viromes based on databases of currently known reference genomes (that are biased towards cultivable organisms) tends to overlook the majority of the community. This disadvantage hampers our capacity to describe and quantify the diversity of viral genomes throughout the marine ecosystem via metagenomics. Assembling viral reads *de novo* to produce sample-specific reference databases has helped to circumvent this issue^24^,^25^,^26^,^27^. Such a strategy improves read mapping and often reveals new complete viral genomes or genome fragments^28^,^29^,^30^.

We sought to expand the knowledge on the genetic diversity of marine viruses by discovering new genomes through a high-throughput culture-independent methodology. To that end, we assembled reads from 78 previously published marine viromes. We discovered new viral lineages derived from highly abundant members of marine viral communities that infect numerically dominant members of the marine microbiome. We then characterized the newly discovered viruses in terms of the diversity of their metabolic genes and predicted which organisms they would infect by using both new and previously validated computational host prediction strategies. With that information, we investigated the distribution profile of these newly discovered sequences across the oceans to further understand how environmental conditions together with microbial host abundances affect the strategies used by marine viruses to exploit their microbial hosts. Our findings corroborate the recently proposed Piggyback-the-Winner theory and demonstrate how viral communities respond to the different seasonal, temperature and photic regimes across the global ocean.

## Results

### Novel diversity from the virome assembly

The assembly of 78 marine viromes (Supplementary Table 1) yielded a total of 27,346 marine virome contigs (MVCs) longer than 2.5 kbp (N50=4,216) that added up to ∼122 Mbp of sequence data. Of these, 44 were circular and longer than 20 kbp and putatively represented complete viral genomes. The remaining contigs were likely fragments of larger genomes or complete linear genomes. Virome reads were randomly subsampled before assembly to allow for longer contigs to be assembled by reducing the genetic microdiversity. This approach successfully improved the assembly quality because the longest version of the majority of contigs was obtained from the subsampled viromes (Supplementary Fig. 1a) with no reduction in the quality of the assembled contigs (Supplementary Fig. 1b). Next, relative abundances of reference viral genomes and MVCs at 121 marine sites (Supplementary Data 1) were calculated as follows. Reads from the 78 selected viromes plus 43 Tara oceans viromes^26^ were aligned to a database containing the MVCs and the reference viral genomes (that is, bacterial and archaeal viruses from the National Center for Biotechnology Information (NCBI) RefSeq database, complete marine phage genomes obtained from fosmid libraries^31^ and prophages identified in bacterial genomes with VirSorter^32^) for a total of 32,833 sequences. Among the reads from 121 analysed marine viromes, 2.2 to 82.5% (average 30.4%, s.d. 17.7%) of them could be assigned to the MVCs. Moreover, 0.06 to 15% (average 4.1%, s.d. 3.42%) of these reads were assigned to reference viral genomes, and 10.2 to 96.7% remained unassigned (average 65.7% s.d. 19.1%). This result provided evidence that the MVCs are highly abundant members of viral communities that outnumbered all currently known prokaryote viral genomes together (Supplementary Fig. 2). The use of the new viral database built with both MVCs and reference viral genomes resulted in a median 6.6-fold increase in read mapping, allowing for up to 82% of virome read annotation. A total of 175,540 proteins were predicted to be encoded by the MVCs, of which 107,260 (61%) appeared to be novel, as no homologues were identified when compared with the NCBI non-redundant (NCBI-nr) database (Supplementary Data 2).

**Figure 1:**
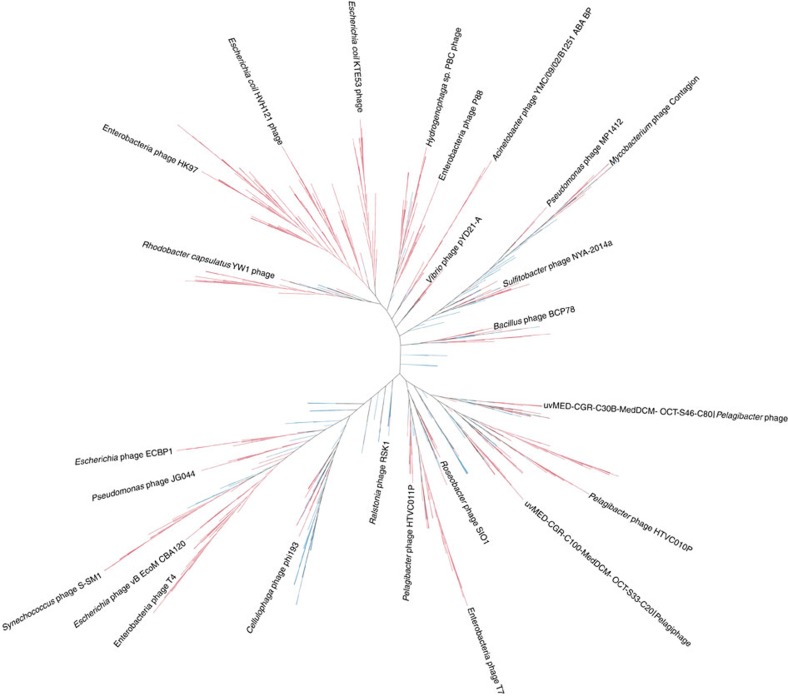
Clustering of the MVCs and the reference phage genomes based on the Dice distances. The MVCs (blue) form novel branches with low similarity to reference phage genomes (red), indicating that they are members of previously unknown lineages of viral diversity. The branch lengths are ignored to better display the clustering topology. Supplementary Data 3 displays a circular version emphasizing exact branch lengths, and Supplementary Data 4 is a circular version that also ignores the branch lengths.

The MVCs and the reference viral genomes were subjected to neighbour-joining clustering on the basis of their Dice distances (see Methods). The MVCs were spread throughout the clusters, suggesting that these newly identified viruses belonged to diverse phylogenetic groups (Fig. 1). Furthermore, several clusters were formed exclusively by MVCs with very long branch lengths that evidenced the low similarity between them and the reference viral genomes (Supplementary Data 3 and 4). This pattern shows that these MVCs are the first members of yet uncharacterized evolutionary viral lineages.

### Phage co-occurrence network and host prediction

**Figure 2:**
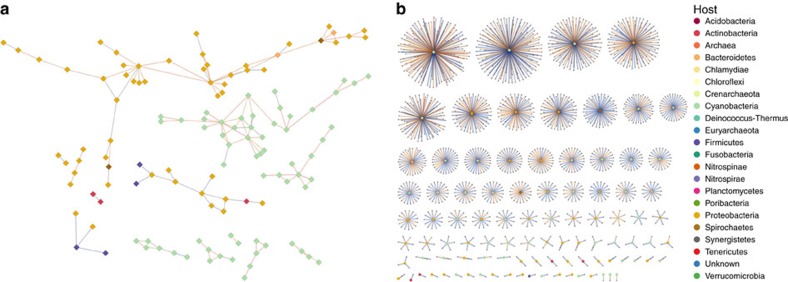
Viral co-occurrence networks. The large diamonds represent the reference viral genomes colour coded according to the host phylum, and the small grey diamonds represent the MVCs. The line colours follow a gradient according to SparCC score from blue (0.6) to red (0.9). (**a**) The network displaying the strongest correlations with a SparCC score >+0.6 between reference phage genomes only. (**b**) The network displaying the strongest correlations with a SparCC score >+0.6 between MVCs and reference phage genomes.

The abundances of each pairwise combination of MVCs and reference viral genomes across samples were correlated with SparCC^33^ to infer a co-occurrence network (Fig. 2). All possible pairwise correlations between the viral genome abundances were assigned a value between −1 and +1. We compared the distribution of the correlation values between the reference viral genomes according to the genus of the host they infect. Correlation values with an absolute SparCC score <0.3 were considered too close to zero for a reliable assessment of their signal and were excluded from this analysis. Out of 5,108 correlations detected between viral genomes that shared a host of the same genus, 4,971 of them were positive (∼97%), while only 137 (∼3%) were negative (Supplementary Fig. 3). Driven by this observation, we next evaluated the capacity of abundance correlations to computationally predict the hosts of the MVCs. The accuracy of this method was assessed by analysing a subset of the network composed only of the reference viral genomes. For each reference viral genome with a known host, we searched for the strongest positive correlation within the network and measured how often that correlation pointed to a virus that infected the same host at the phylum level. This resulted in ∼57% accuracy if no correlation score cutoffs were used, that is, any value between −1 and 1 was considered a host prediction, as long as the correlation was the highest for that genome (the weakest of these correlations was close to +0.25). Varying the minimum correlation score cutoff revealed that the accuracy of the host predictions could be increased to ∼87% if only scores above 0.6 were considered, although at the expense of predicting fewer hosts. This approach could be applied to host prediction at deeper taxonomic levels (Supplementary Fig. 4a), but with less accurate results (Supplementary Fig. 4b). Using the +0.6 cutoff, we were able to assign hosts to 1,279 MVCs (Table 1 and Supplementary Data 5), most of which were predicted to be Cyanophages that infected *Prochlorocccocus* or *Synechococcus* and Pelagiphages, and some were predicted to infect *Flavobacterium* and *Puniceispirillum*. The majority of the top correlation scores used to assign the hosts to the MVCs were greater than +0.6 (Supplementary Fig. 3); therefore, we assumed that they were accurate at the phylum level.

Correlation network-based host predictions for the MVCs were complemented by four other computational strategies (Table 1 and Supplementary Data 5). Homology matches against a database of bacterial genomes resulted in 268 predictions. The most frequent host predictions obtained via this approach were *Sphyngopyxis* (Alphaproteobacteria), followed by *Propionibacterium* (Actinobacteria) and *Synechococcus* (Cyanobacteria). Homology matches against a database of annotated Tara oceans microbial contigs yielded 1,393 predictions. The most common host predictions were to unclassified Alphaproteobacteria, followed by Verrucomicrobia, Bacteroidetes and Actinobacteria. CRISPR (clustered regularly interspaced short palindromic repeats) spacers mined from bacterial genomes could be linked to 20 MVCs, the majority of which were derived from Proteobacteria genomes (most often from *Xanthomonas*). Through transfer RNA (tRNA) matches, 87 MVCs could be assigned to a host, most frequently to genera that belong to either Proteobacteria or Bacteroidetes. A total of 2,755 MVCs could be assigned to a host by at least one of these five methods (Table 1).

### MVCs are ubiquitous and abundant across the oceans

**Figure 3:**
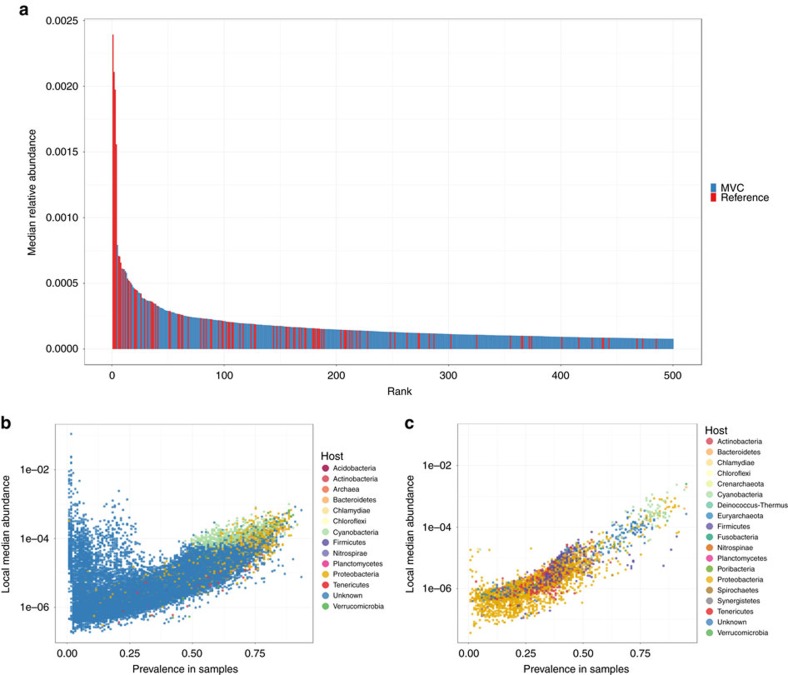
The abundance patterns of the MVCs and the reference viral genomes across 121 marine viromes. (**a**) The rank abundance curve of the top 500 most abundant reference viral genomes and MVCs. (**b**) The *x* axis shows the prevalence (percentage of samples in which an MVC was detected), while the *y* axis shows the median relative abundance of such MVCs across the 121 marine virome samples analysed. MVCs are colour coded according to their predicted host phylum. (**c**) The same as in **b** but displaying the prevalence and median relative abundance of the reference phage genomes, colour coded according to the phylum of their hosts. Supplementary Data 6 displays the average abundance and prevalence of all MVCs and reference phage genomes across the 121 samples.

The rank abundance curve (Fig. 3a) revealed that although reference genomes ranked first, MVCs are among the most abundant members of marine viromes (that is, the top 500). An analysis of the distribution patterns of MVCs across marine virome samples according to their predicted hosts revealed that the most prevalent (detected in >50% of the samples) and abundant (median relative abundance >0.01%) MVCs were those predicted to infect Cyanobacteria and Proteobacteria (Fig. 3b and Supplementary Data 6). This trend was also observed for the reference viral genomes, as the most abundant and prevalent ones infected *Pelagibacter* (Alphaproteobacteria) or *Prochlorococcus* and *Synechococcus* (Cyanobacteria) (Fig. 3c).

### Functional content of viruses varies according to the host

We analysed the functional content of the MVCs and the reference viral genomes according to their infected hosts (Supplementary Data 7). The genes involved in purine/pyrimidine metabolism and nucleic acid biosynthesis were among the most common traits for all viruses. Differences between the host groups were commonly found as potential auxiliary metabolic genes and metabolic or transcriptional regulators. Viruses that infect Cyanobacteria typically encode proteins involved in photosynthesis (that is, photosystem II and plastocyanin), the pentose phosphate pathway and genes involved in carbon, sugar and amino acid metabolism. Moreover, transcriptional regulators and ABC (ATP-binding cassette) transporters are included among the genes most often identified in the genomes of the viruses that infect Proteobacteria. These transporters were also commonly found in the genomes of viruses that infect Firmicutes, but transcriptional regulators were not as prevalent as in the previous group. Finally, viruses infecting Actinobacteria or Bacteroidetes often harboured proteins involved in amino acid metabolism, while the latter also harboured several proteins involved in sugar metabolism.

### Comparison of global marine viral communities

**Figure 4:**
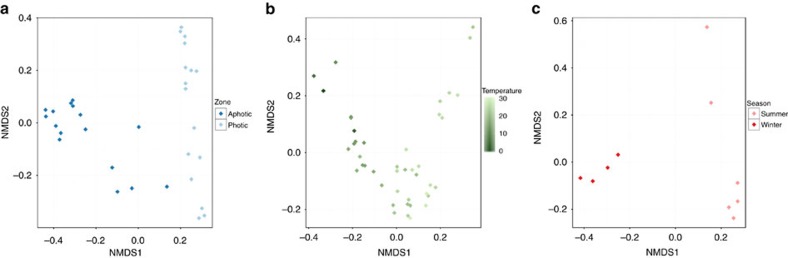
Virome nonmetric multidimensional scaling. The Manhattan distances were calculated based on the viral genome relative abundances and used as the input for a NMDS analysis. (**a**) POVs from photic (light blue) and aphotic (dark blue) zones. (**b**) Tara oceans viromes from warm (light green) and cold (dark green) waters. (**c**) Abrolhos viromes from summer (light red) and winter (dark red) seasons.

We applied nonmetric multidimensional scaling (NMDS) to reveal the clustering patterns of marine viromes based on the abundance of MVCs and reference viral genomes in each sample. The viromes were separated into three data sets to avoid potential clustering resulting from sample preparation biases^34^. The Pacific Ocean viromes (POVs) that were retrieved from a broad depth gradient across three sites in the Pacific were separated between photic and aphotic zone samples by NMDS1 (Fig. 4a). Tara oceans viromes, a data set of photic zone samples obtained across the global oceans, did not cluster according to geographical location. Therefore, the NMDS axis values were correlated with the environmental parameters measured at the sampling sites. Temperature yielded the strongest Spearman’s correlation coefficient (0.89) to NMDS1, followed by *Prochlorococcus* cell abundance (0.63). Thus, the Tara oceans viromes were separated by NMDS1 into two major groups according to water temperature (Fig. 4b). Finally, the Abrolhos samples from warm water coral reef environments of the photic zone were separated between summer and winter viromes (Fig. 4c).

### Shifts in viral communities with environmental conditions

The abundance profiles of the marine viromes were used to identify viruses whose abundance differed significantly between the sample groups identified through NMDS. The viromes were divided into three group pairs: POV Aphotic (>500 m deep) × POV Photic (<105 m deep); Tara Cold (<23.3 °C) × Tara Warm (>23.3 °C); and Abrolhos Summer × Abrolhos Winter. Supplementary Table 2 lists the groups to which each sample was assigned. The abundance of each MVC and the reference viral genome between the sample groups was compared using the Mann–Whitney test, followed by correction for multiple testing via the false discovery rate^35^. Significant changes in abundance (that is, a corrected *P* value of <0.05) in at least one of the sample groups were detected for a total of 7,614 MVCs and reference viral genomes (Supplementary Data 8).

**Figure 5:**
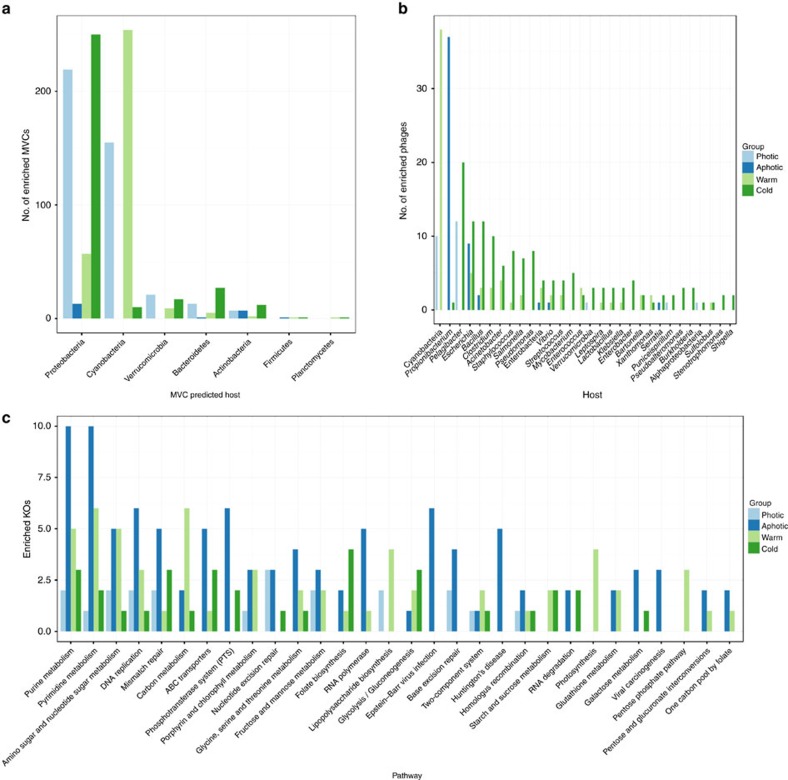
Variables displaying significant changes in abundance across sample groupings. The bar lengths (*y* axis) are proportional to the number of variables in a given category (*x* axis) enriched in each of the tested sample groupings (that is, photic, aphotic, warm and cold) as determined by the Mann–Whitney test (corrected *P* value <0.05). (**a**) Enriched MVCs grouped according to the predicted host phylum. (**b**) Enriched reference viral genomes grouped according to the known host genus. Cyanobacteria refers to viruses that infect *Prochlorococcus* and *Synechococcus*. (**c**) Enriched KOs grouped according to the metabolic pathways to which they belong.

Mann–Whitney tests revealed that the POV Photic zone had significantly higher abundances of MVCs predicted to infect Cyanobacteria (a total of 155 MVCs most often predicted to infect *Prochlorococcus* or *Synechococcus* were enriched in these samples) or Proteobacteria (219, including *Pelagibacter*, *Puniceispirillum* and many unclassified members of this phylum). Meanwhile, the POVs from aphotic zone samples had significantly higher abundances of MVCs predicted to infect Proteobacteria (13) or Actinobacteria (7) such as *Vibrio* and *Propionibacterium*. The Tara viromes obtained from warm water sites had significantly higher abundances of MVCs predicted to infect Cyanobacteria (254 in total, mainly predicted to infect *Prochlorococcus* or *Synechococcus*) or Proteobacteria (57 in total, predicted to infect mostly unclassified members of this phylum) and, finally, the most often enriched MVCs from cold water sites were predicted to infect Proteobacteria (250, mostly unclassified followed by *Pelagibacter*, *Puniceispirillum*) and Bacteroidetes (27, most often *Flavobacterium*) (Fig. 5a).

The reference viral genomes corroborated the enrichment trends observed for the MVCs (Fig. 5b). The reference viral genomes that targeted Cyanobacteria or Alphaproteobacteria (for example, *Pelagibacter* and *Puniceispirillum*) were enriched in POVs from the photic zone, while the aphotic zone samples were enriched for viruses that infected chemoheterotrophic bacteria such as *Propionibacterium* and *Escherichia*. The cyanophages were the most common reference viral genomes enriched at warm water Tara viromes. In contrast, Pelagiphages and other viruses that infect chemoheterotrophic bacteria were enriched at cold water Tara viromes.

The viromes were also compared according to their functional profiles, that is, the relative abundances of KEGG (Kyoto Encyclopedia of Genes and Genomes) orthologues (KOs) in each sample. A total of 297 KOs present in the MVCs or the reference viral genomes showed significant (that is, a corrected *P* value of <0.05) differences in abundance between the sample groups tested (Supplementary Data 9). When compared with their photic counterparts, the POVs from the aphotic zone samples were characterized by the enrichment of KOs including those involved in nucleic acid metabolism pathways (for example, purine and pyrimidine metabolism and DNA replication) and ABC transporters. Moreover, a comparison of cold water against warm water Tara viromes revealed that the latter were characterized by the enrichment of KOs including those involved in carbon metabolism, photosynthesis, lipopolysaccharide biosynthesis and the pentose phosphate pathway (Fig. 5c).

### The virus/host ratio and host abundance correlate negatively

**Figure 6:**
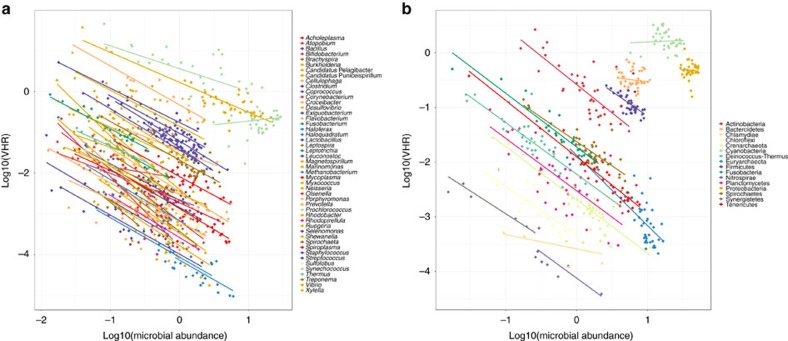
Associations between the microbial host abundance and the virus–host ratio. The *x* axis displays the abundances of microbial taxa and the *y* axis displays VHR, calculated based on the relative abundances of microbial taxa and the viruses that infect them in the analysed Tara oceans microbial metagenomes and viromes. (**a**) Microbial taxa are summed at the taxonomic levels of genus and VHR was calculated using the abundances of reference viral genomes only. (**b**) Microbial abundances are summed at the taxonomic level of phylum and VHR was calculated using the abundances of both reference viral genomes and the MVCs for which a putative host was identified.

We compared the relative abundance of the viral genomes with that of their microbial hosts in paired viromes and metagenomes from the Tara oceans data set. The virus/host ratio (VHR, defined as the viral genome abundance divided by the host genome abundance) was negatively correlated with the host abundance at the levels of genus (Fig. 6a and Supplementary Table 3, reference viruses only) or phylum (Fig. 6b and Supplementary Table 4, reference viruses and MVCs with host prediction).

## Discussion

The MVCs included novel viral genomes and genome fragments. These sequences were divergent from previously known viral genomes as evidenced by their very long branch lengths (Supplementary Data 4). This result draws attention to the major gap in our knowledge regarding the diversity of marine viruses. In this study, we closed that gap by discovering new marine viruses without the use of culture- and isolation-based approaches to directly obtain complete viral genomes from marine viromes. The discovery of the MVCs and other viruses via metagenomics helps to characterize new viral lineages that were overlooked by culture-dependent methods^29^,^31^,^36^,^37^. These new genomes will improve our understanding of the processes of viral diversification and evolution. Additionally, including the MVCs in the reference database allowed for a more comprehensive characterization of marine viral communities via metagenomics.

A co-occurrence network analysis was applied to investigate the associations between microorganisms. When organisms use the same resources and respond similarly to environmental factors, their abundances are expected to be positively correlated^38^,^39^,^40^. Viruses depend on a host to successfully replicate. Therefore, the virus and host abundance across spatial and temporal gradients are generally associated^12^,^41^,^42^,^43^,^44^. Viruses that target the same organism compete for a host when present at the same site simultaneously. Positive correlations were dominant among viruses that targeted hosts of the same genus (Supplementary Fig. 3). The observed strong positive correlation trend between competitors allows co-occurrence networks to be used as a new host prediction method. Negative correlations between viruses that shared the same host were also detected (Supplementary Fig. 3). Because this type of association was very rare within the network, they were not used for host prediction but they could have resulted from the competitive exclusion between viruses that shared the same host and thus also have potential to be used for host prediction. Co-occurrence between viral and bacterial abundance has been suggested as a host prediction method, but with a low predictive capacity^45^. To the best of our knowledge, this is the first time that virus–virus abundance associations were used for host affiliation. The method performed well for host prediction from the phylum to the genus level (Supplementary Fig. 4) and yielded nearly 50% of all of our host predictions (Table 1). Furthermore, this approach was not dependent on the detection of exchanges of genetic material between viruses and their hosts as required by homology matches and CRISPR.

An analysis of paired viral and microbial Tara oceans metagenomes^24^,^26^ indicated a reduction in the VHR towards higher host abundances (Fig. 6). Assuming an increase in sequence abundance proportional to the cell and viral particles abundance in the environment, we predict a decrease in the specific host/virus pairs ratio with an increased host abundance. This pattern corroborates the decrease in VHR with an increase in microbial abundance described by the Piggyback-the-Winner model and hypothesizes lysogeny as a more successful strategy for viral replication at a high host density^11^. The negative relationship between the host and viral abundance emerged consistently in the majority of the ecosystems studied^11^,^12^, and habitats with increased prokaryotic abundance were also enriched for markers of lysogenic infection (for example, integrases or excisionases)^11^. Our data corroborated the Piggyback-the-Winner model by using a completely independent data set and demonstrated the ubiquity of this trend for nearly all the detected taxa of microorganisms (Supplementary Tables 3 and 4).

The pattern observed could be explained by a model in which the viruses opt for a lysogenic infection strategy when their microbial hosts are thriving (that is, at high abundance). Recent findings showed that prophages are widespread in prokaryote genomes, including those taxa that are dominant across marine habitats (for example, Cyanobacteria, Proteobacteria, Firmicutes, Bacteroidetes and Actinobacteria)^32^ and that fast-growing bacteria are more likely to harbour prophages integrated into their genomes^46^,^47^. Finally, the observed reduction in the ratio between bacterial cells and viral particles at increased microbial abundances was consistently reported across marine ecosystems^11^,^12^. At high host densities, rather than killing their hosts, viruses might opt to replicate integrated into their host genomes. According to this model, whenever conditions change and host growth is no longer favoured, the virus goes into a lytic cycle to ensure the production of new viral particles before the death of the host makes viral replication impossible. A total of 134 MVC proteins were annotated as integrases or excisionases (Supplementary Data 2), providing further evidence for the capacity of lysogenic infections among the MVCs.

Other factors can act in association with lysogenic switching and result in the observed trend of decrease in the VHR accompanied by an increase in microbial abundance. Although our previous analysis detected no association between resistance mechanisms (for example, CRISPRs) and microbial abundance^11^, the dissemination of resistant strains might contribute to the aforementioned trend. This might be the case especially for some slow-growing marine bacteria whose genomes do not encode prophages (for example, *Pelagibacter*, *Puniceispirillum* and *Synechococcus*^32^). This is not proof that lysogenic viruses do not infect these organisms, but it does suggest that for some taxa, the negative association between VHR and host abundance might be driven by both lysogenic switching and resistance to viral infection.

Use of the MVCs together with reference phage genomes allowed us to identify differences in the genomic composition of viruses according to their infected hosts (Supplementary Data 7). We also identified significant differences in the viral community taxonomic and functional composition across environmental gradients, namely photic/aphotic and warm/cold habitats (Fig. 5). Taken together, these results clarify how the viral community composition adapts according to the host community composition to better exploit the host communities. The marked shift in the community composition among these habitats was also observed in our NMDS analysis of microbial metagenomes (cellular fraction) across depth and temperature gradients (Supplementary Fig. 5). Furthermore, the viruses and their hosts displayed consistent enrichment patterns (including dominant marine taxa such as *Pelagibacter*, *Prochlorococcus* and *Synechococcus*) when comparing photic/aphotic and warm/cold samples (Supplementary Data 8 and 10). Considering these results together with the viral dependence on the host metabolism for replication, we concluded that the differences we identified in the viral community composition were derived from the modulation of the metabolism and growth rates of the microbial hosts as by environmental conditions. Thus, the viral communities were indirectly affected by the photic/aphotic and warm/cold water regimes^48^. We could not determine the individual effect of each of the many environmental parameters (for example, temperature, nutrients, microbial growth rates and so on) that characterize these habitats on the modulation of the viral and microbial community composition. Therefore, we assumed that the observed shifts in the microbial and viral communities were a result of their combined effects. Interestingly, light emerged as a major factor that regulated the viral community composition that could be linked not only to the differences between the photic and aphotic habitats but also to the distinction between the warm/cold and the summer/winter samples because the water temperature is influenced by the degree of solar irradiance that in turn oscillates between the seasons.

**Figure 7:**
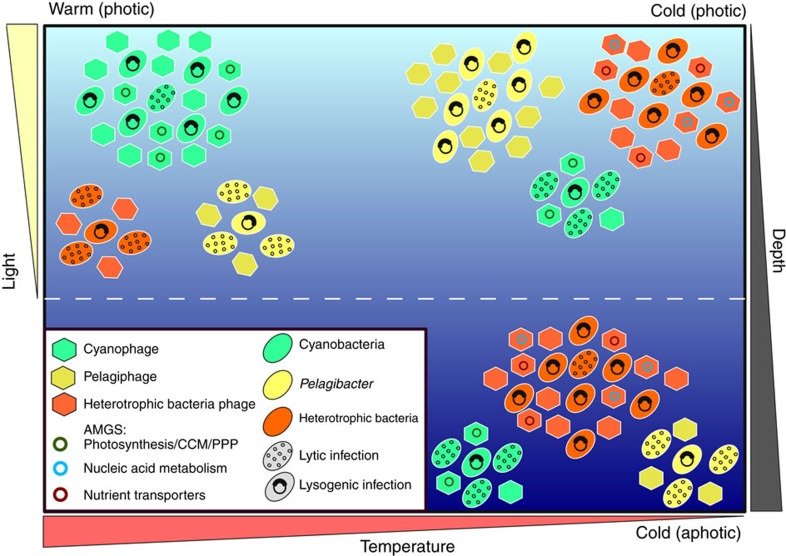
Conceptual model depicting viral strategies for exploiting the marine microbiome. In the warm waters of the photic zone, Cyanophages would be enriched and display a preference for lysogenic infections. Under these same conditions, Pelagiphages and viruses infecting heterotrophic bacteria would be depleted and prefer lytic infections. In the cold waters of the photic zone, the opposite pattern would occur: Cyanophages depleted and lytic, and Pelagiphages and viruses infecting heterotrophic bacteria would be enriched and lysogenic infections. In the cold waters of the aphotic zone, both Cyanophages and Pelagiphages would be depleted and lytic, while viruses infecting heterotrophic bacteria would be enriched and lysogenic. Throughout these gradients, these viruses carry different types of auxiliary metabolic genes that help them to exploit host metabolism during infection.

Cyanophages and Pelagiphages were found to be enriched in photic zone viromes, while phages infecting chemoheterotrophic bacteria (for example, *Vibrio* and *Propionibacterium*) were enriched in aphotic zone viromes (Fig. 5a,b and Supplementary Data 8). The abundance of organisms that rely on light-dependent mechanisms for energy acquisition such as Cyanobacteria and *Pelagibacter* was smaller in aphotic regions dominated by chemoheterotrophic bacteria^38^,^49^,^50^,^51^. This shift in the composition of host bacterial community explains the enrichment patterns observed for the viral fraction. In the deep ocean, light becomes unavailable, and temperature, organic carbon availability and primary productivity decrease, leading to lower bacterial growth rates^51^,^52^. Those conditions likely favour viral communities that encode auxiliary metabolic genes that modulate bacterial metabolism towards pathways that facilitate viral replication under conditions that tend to slow down microbial metabolism. For example, the aphotic zone samples were enriched for several KOs associated with ABC transporters and nucleotide synthesis (Fig. 5c). These genes might be used in mechanisms by which viral communities enhance bacterial nutrient uptake and nucleotide synthesis rates to ensure the availability of building blocks required for the synthesis of new viral particles under nutrient-deprived conditions^14^,^18^ (Fig. 7).

Warm water samples were enriched in viruses that infected *Prochlorococcus* and *Synechococcus*, while those that infected *Pelagibacter*, *Puniceispirillum*, *Flavobacterium* and other heterotrophic bacteria were typically enriched in cold water habitats (Fig. 5a,b and Supplementary Data 8). The increase in the abundance of Cyanobacteria driven by higher temperatures explains the enrichment of Cyanophages in warm waters^26^,^38^,^53^,^54^. These samples were also enriched in many KOs involved in photosynthesis, carbon metabolism and the pentose phosphate pathway (Fig. 5c), suggesting that viral communities from warm waters with a higher abundance of Cyanobacteria exploit the photosynthetic microbial community by modulating photosynthesis and carbon fixation towards pathways that favour the synthesis of viral particles^15^,^17^. Moreover, in cold water, the viruses tend to rely more on infecting nonphotosynthetic organisms and modulating their heterotrophic metabolism (Fig. 7).

Metagenomics-based studies have previously investigated shifts in the viral community composition driven by environmental parameters, but did so through annotation independent (k-mer based) or protein cluster-based analyses^14^,^55^,^56^. Using our improved database for virome annotation that includes the highly abundant MVCs allowed us to corroborate and expand these results. Unlike k-mers or protein clusters, MVCs carry associated information regarding their sampling source, host and the complete or partial genomes of the viruses from which they are derived. This allows for a more comprehensive understanding of the differences in the community composition of the sample groups tested that in turn could be linked to the environmental conditions.

In conclusion, we have described and analysed over 27,000 MVCs, a unique data set of complete and partial marine viral genomes derived from highly abundant members of global marine viromes. Many of these viruses belong to completely novel lineages. Computational host prediction, including a new accurate approach based on viral co-abundance correlations, suggests that most MVCs infect dominant marine bacteria including Cyanobacteria and Proteobacteria. We showed that for practically all taxonomic groups, a negative association was present between the host relative abundance and VHR, suggesting that more lysogeny and possibly resistance occurred at higher relative host densities and was a widespread trend among marine viruses and their hosts. Finally, the global distribution of the MVCs revealed how marine viral communities adapt their composition and diversity of auxiliary metabolic genes to exploit their microbial hosts according to changes in depth, temperature and season. The findings presented here, together with recent discoveries made on the ecology of marine viruses based on metagenomics^13^,^31^,^55^,^56^,^57^,^58^,^59^, shed light on the poorly explored marine viral diversity and bring us closer to understanding the role of viruses in the function of marine ecosystems.

## Methods

### Virome samples and assembly

A total of 78 previously published and quality-controlled marine viromes (that is, post read trimming and filtered for low-quality sequences and potential contaminants) were selected from Metavir^60^ in March 2015. These viromes were obtained from marine habitats, including photic and aphotic regions of coastal and open ocean regions, oxygen minimum zones, coral reef systems and coral holobionts. Supplementary Table 1 describes these viromes in terms of the number of sequences, the average sequence length and their original publication. Virome assemblies were performed via a random subsampling approach aimed at obtaining longer contigs by reducing the microdiversity within the samples. Large amounts of sequencing errors or microdiversity can lead to fragmented assemblies^61^,^62^. An analysis of the effects of the coverage depth on the virome assembly quality revealed that viral genomes can often be oversequenced, that is, the coverage is extremely high but so are the errors, leading to fragmented assemblies, a phenomenon that can be avoided by using a smaller data set that has fewer sequences but also fewer errors, consequently improving the assembly quality^61^. Subsampling was expected to facilitate the assembly of sequences derived from the most abundant members of the community at the expense of increasing the difficulty of the assembly of the less abundant sequences. Therefore, each member of the community should have an optimum number of reads for the best assembly with maximum coverage and minimum error. Our assembly strategy was designed to achieve an optimum range of reads for as many sequences as possible. We aimed to obtain the best assemblies possible (through the use of different subsample sizes) while avoiding the loss of diversity due to random subsampling by repeating several assemblies for each subset. Our strategy was based on the random selection of a subset of the reads from each sample (ranging from 1 to 100%) and then assembling these subsets individually. Viromes containing <100,000 reads were subsampled to 25% of the reads (repeated 20 times), 50% (10 × ), 75% (10 × ) and 100% (1 × ). Viromes containing 100,000 to 1,000,000 reads were subsampled to 10% (50 × ), 25% (25 × ), 50% (25 × ), 75% (20 × ) and 100% (1 × ). Viromes containing >1,000,000 reads were subsampled to 1% (75 × ), 5% (50 × ), 10% (50 × ), 25% (25 × ), 75% (25 × ) and 100% (1 × ) of the data. In addition, four cross-assemblies were performed that merged all of the reads from samples of the Pacific Ocean Viromes, Abrolhos coral reefs, oxygen minimum zones and Indian Ocean data sets. These merged data sets were subsampled and reassembled using the same strategy described above according to the number of reads in each. The assemblies were performed by IDBA_UD^63^ using the default parameters and pre-correction. Contigs derived from all of the assemblies were combined, and those <2,500 bp were removed. BLASTn was used to dereplicate the contigs, using an identity cutoff of 95% and a minimum alignment coverage of 40% of the shorter sequence. The resulting database of non-redundant Marine Virome Contigs is available at http://www.ebi.ac.uk/ena/data/view/PRJEB19352. Coding DNA sequences were identified with Prodigal^64^ within Prokka^65^. Protein sequences were queried against the NCBI NCBI-nr database for annotation using Diamond^66^, setting a maximum *e*-value of 10^−5^ and a minimum identity of 40%.

### Genome comparisons

We focused our analysis on bacterial and archaeal viruses (phages) because they are the numerically dominant members of marine viral communities^26^. A database of known phage genomes was built by merging the MVCs with a set of reference viral genomes obtained from three sources: (1) the NCBI RefSeq database (1,609 sequences); (2) the complete marine phage genomes obtained from fosmid libraries (208)^31^ and (3) prophages identified in bacterial genomes with VirSorter (12,498)^32^. The database was made non-redundant by clustering the genomes with BLASTn with a 95% identity and a 40% coverage cutoff, resulting in a non-redundant data set of 32,833 sequences. Next, the Dice coefficient score was used to estimate the distances between the MVCs longer than 20 kbp and the reference viral genomes to organize them into a phylogenomic framework^31^. This approach was selected because it allowed for the degree of similarity between phage genomes to be estimated without the need for multiple alignments or the clustering of sequences into homologue groups or the use of universal marker genes, all of which are major disadvantages for the unbiased investigation of viral phylogeny^67^. Only reference viruses that had at least one detectable homologue to MVCs as determined by tBLASTx^68^ searches were used for this analysis. The Dice distance calculation was based on an all-versus-all tBLASTx search between the viral genomes. Any hits that either scored <30% identity, were shorter than 30 amino acids or had an *e*-value >0.01 were ignored. The distances between the viral genomes or MVCs were measured as D_A,B_=1−(2 × AB/AA+BB), where AB is the summed bitscore of all hits of genome A against genome B. AA and BB represent the summed bitscore of all hits of genomes A and B against themselves. The obtained distance matrix was used to cluster the genomes via neighbour joining by the BIONJ^69^ algorithm, and visualized in iTOL (Interactive Tree Of Life)^70^.

### Abundance profiles

A matrix of abundances of all of the MVCs at 121 marine sites was calculated as follows. Reads from the 78 selected viromes plus 43 Tara oceans viromes^26^ were mapped against the database of viral genomes using Bowtie2 (ref. 71). The very-sensitive alignment option was used along with read end trimming and multiple matching to maximize the read mapping. Ambiguous reads that were mapped to similar regions of different genomes were counted using a weighted score based on the ratios of the unambiguous reads assigned to each genome as previously described^72^.

### Network inference

An abundance matrix was used to infer correlations between viral genome abundances across samples. The SparCC method was applied to avoid spurious correlations that emerged from the sparse and compositional nature of the data^33^. Any MVC or reference genome detected in <40% of samples was excluded from this analysis because these have been shown to lead to spurious correlations due to sparse counts^73^. SparCC was run with 10 inference and 10 exclusion iterations. The resulting network of correlations was visualized with Cytoscape^74^.

### Host predictions

We used multiple computational host prediction strategies to identify potential microbial hosts infected by the MVCs^45^. (1) Homology matches against bacterial and archaeal genomes: the MVCs were queried against a database of microbial genomes obtained from NCBI through BLASTn. Only the best hits above 80% identity across an alignment of at least 1,000 nucleotides were considered. (2) The aforementioned database of bacterial genomes is biased towards cultured organisms that do not necessarily represent the diversity of prokaryotes abundant in the oceans. To circumvent this issue, we also performed homology matches of the MVCs against the Tara oceans contigs obtained from http://www.ebi.ac.uk/ena/about/tara-oceans-assemblies^24^. This data set is a large catalogue of marine microbial sequences that, similar to our MVCs, were obtained via culture-independent methods and from several regions of the global oceans. First, the Tara oceans contigs were taxonomically annotated by predicting protein sequences by Prodigal and querying them against the NCBI-nr database using Diamond. Only the best hits of each protein with an *e*-value <10e^−5^ and an identity >30% were considered. Next, the sum of the bitscore of all hits from each contig was calculated, and the contigs for which the total bitscore was below 1,000 were disregarded. A hierarchical classification of the remaining contigs was performed from domain to species if 80% or more of the total bitscore was consistently assigned to the same taxon. The contigs unclassified at the domain level or classified as viral or eukaryotic were excluded. (3) CRISPR spacers within the microbial genomes were identified using CRISPR Detect v.1. Those spacers were queried against the MVCs using the BLASTn parameters described in ref. 75. Because CRISPR spacers are very short sequences (∼20–30 nucleotides), a maximum of two mismatches/gaps was allowed to minimize the chances of erroneous host assignments due to spurious matches. (4) tRNA matches: transporter RNAs identified in MVCs were queried against a database of bacterial genomes using BLASTn and only the best hits with a minimum of 90% identity and 90% coverage were considered. (5) Abundance correlations: we developed a new strategy for host prediction based on abundance correlations between the MVCs and the reference phage genomes across the marine viromes. The MVCs were assigned to a host based on the strongest positive correlation with a reference viral genome. Only those correlations that fell within an experimentally defined cutoff (SparCC score ≥+0.6) were considered to maximize the number of accurate MVC host assignments (see the Results section ‘Phage co-occurrence network and host prediction’ for further details).

### Functional profiles

All proteins encoded by the MVCs and the reference phage genomes were queried against the OM-RGC database^24^ via Diamond^66^ and annotated according to the KOs to which their best hit was assigned (maximum *e*-value of 10^−5^). Next, the functional profiles (that is, the KO relative abundances) were determined for each sample by summing up the abundance of each KO proportionally to the abundance of the genome or the MVC in which it was encoded. For example, in a sample containing genomes A, B and C with abundances of 1, 5 and 10, the KO abundance in that sample would be defined as the sum of KOs encoded in A multiplied by 1, plus those encoded in B multiplied by 5 and those encoded in C multiplied by 10.

### Marine microbial community analysis

We reanalysed the microbial marine metagenomes first to compare the effects of environmental parameters on the viral and microbial fractions of the marine ecosystems. Second, we wanted to determine how the viral abundances were associated with those of the microbial hosts they infect. To that end, the microbial metagenomes (cellular fraction) that covered a broad spatial range and gradients of environmental parameters were selected. The Tara oceans metagenomes^24^ were analysed to investigate microbial community composition across a broad spatial gradient. The South Atlantic Ocean (SAO) metagenomes^76^ covered both the photic and aphotic zones within this region of the ocean. The abundance of the bacterial and archaeal genomes in both the Tara and SAO metagenomes was modelled based on the nucleotide composition profile using FOCUS with k-mer size of seven nucleotides^77^.

### Nonmetric multidimensional scaling

Both the virome and microbial metagenome samples were compared on the basis of their taxonomic composition profiles. The distances between samples were calculated based on the Manhattan method and used as the input for NMDS. To avoid clustering driven by sampling preparation biases^34^, these analyses were performed separately for subsets of samples that were consistent in terms of their processing methodology: POVs, Tara oceans and Abrolhos viromes and for Tara and SAO microbial metagenomes.

### Variable enrichments

The microbial metagenomes and viromes were grouped according to their NMDS clustering patterns (Supplementary Table 2). Next, the relative abundances of each viral genome/MVC, KO or microbial taxon found in the metagenomes and viromes were compared between sample groups using the Mann–Whitney test. The *P* values were corrected for multiple testing via the false discovery rate^35^, and differences in abundance that yielded a corrected *P* value of <0.05 were considered significant.

### Data availability

All sequences assembled from the 78 marine viromes were deposited at ENA: http://www.ebi.ac.uk/ena/data/view/PRJEB19352.

## Additional information

**How to cite this article:** Coutinho, F. H. *et al*. Marine viruses discovered via metagenomics shed light on viral strategies throughout the oceans. *Nat. Commun.* **8,** 15955 doi: 10.1038/ncomms15955 (2017).

**Publisher’s note:** Springer Nature remains neutral with regard to jurisdictional claims in published maps and institutional affiliations.

## Supplementary Material

Supplementary Information

Supplementary Data 1

Supplementary Data 2

Supplementary Data 3

Supplementary Data 4

Supplementary Data 5

Supplementary Data 6

Supplementary Data 7

Supplementary Data 8

Supplementary Data 9

Supplementary Data 10

## Figures and Tables

**Table 1:** The number of MVCs assigned to each host taxa according to the five host prediction methods.

	**RefSeq homology**	**Tara homology**	**CRISPR**	**tRNA**	**Network**
Unclassified Proteobacteria	0	868	0	0	0
*Prochlorococcus*	10	0	0	0	575
*Pelagibacter*	0	0	0	1	461
*Synechococcus*	8	2	0	4	146
*Sphingopyxis*	136	0	0	11	0
*Flavobacterium*	0	82	0	1	59
Unclassified Verrucomicrobia	0	142	0	0	0
Unclassified Actinobacteria	0	76	0	0	0
*Propionibacterium*	52	0	0	2	4
*Puniceispirillum*	0	3	0	1	17
*Bradyrhizobium*	0	16	0	0	0
*Blastomonas*	0	15	0	0	0
Unclassified Alphaproteobacteria	0	12	0	0	0
*Sphingobium*	7	3	0	2	0
*Acidovorax*	10	0	0	1	0
*Desulfovibrio*	0	10	0	0	0
*Pseudomonas*	3	2	2	0	2
*Burkholderia*	6	0	2	0	0
*Xanthomonas*	1	0	7	0	0

Only the top 20 most frequent taxa are shown. Supplementary Data 5 details the host predictions and the scores yielded by each method per MVC.
