# Supplementary material for: Marine viruses discovered via metagenomics shed light on viral strategies throughout the oceans
Source: Nat Commun. 2017 Jul 5;8:15955. doi: 10.1038/ncomms15955 (PMC5504273; doi:10.1038/ncomms15955)
Supplement: Supplementary Information [file ncomms15955-s1.pdf]

File Name: Supplementary Information
Description: Supplementary Figures, Supplementary Tables and Supplementary References

File Name: Supplementary Dataset 1
Description: Abundance profile of the MVCs and the reference phage genomes (rows) across the 121 marine viromes (columns).

File Name: Supplementary Dataset 2
Description: Functional and taxonomic annotation of the proteins encoded by the MVCs compared to the NCBI-nr database.

File Name: Supplementary Dataset 3
Description: Circular neighbour-joining clustering of the MVCs (blue) and the reference phage genomes (red) based on Dice distances depicting the true branch lengths.

File Name: Supplementary Dataset 4
Description: Circular neighbour-joining clustering of the MVCs (blue) and the reference phage genomes (red) based on Dice distances with the branch lengths omitted for better topology representation.

File Name: Supplementary Dataset 5
Description: A summary of all 5 approaches used for host prediction and their respective scores for each MVC.

File Name: Supplementary Dataset 6
Description: The statistics of the abundance profiles of the MVCs and the reference viral genomes across the 121 marine viromes.

File Name: Supplementary Dataset 7
Description: Functional content derived from the MVCs and the reference phage genomes. The values represent the average number of occurrences of the KO in each group of phages according to the host phylum.

File Name: Supplementary Dataset 8
Description: A list of the MVCs and the reference phages enriched or depleted in abundance among the tested samples groups.

File Name: Supplementary Dataset 9
Description: A list of the KOs enriched or depleted in abundance among the tested samples groups.

File Name: Supplementary Dataset 10
Description: A list of the bacterial and archaeal species enriched or depleted in abundance among the tested samples groups.

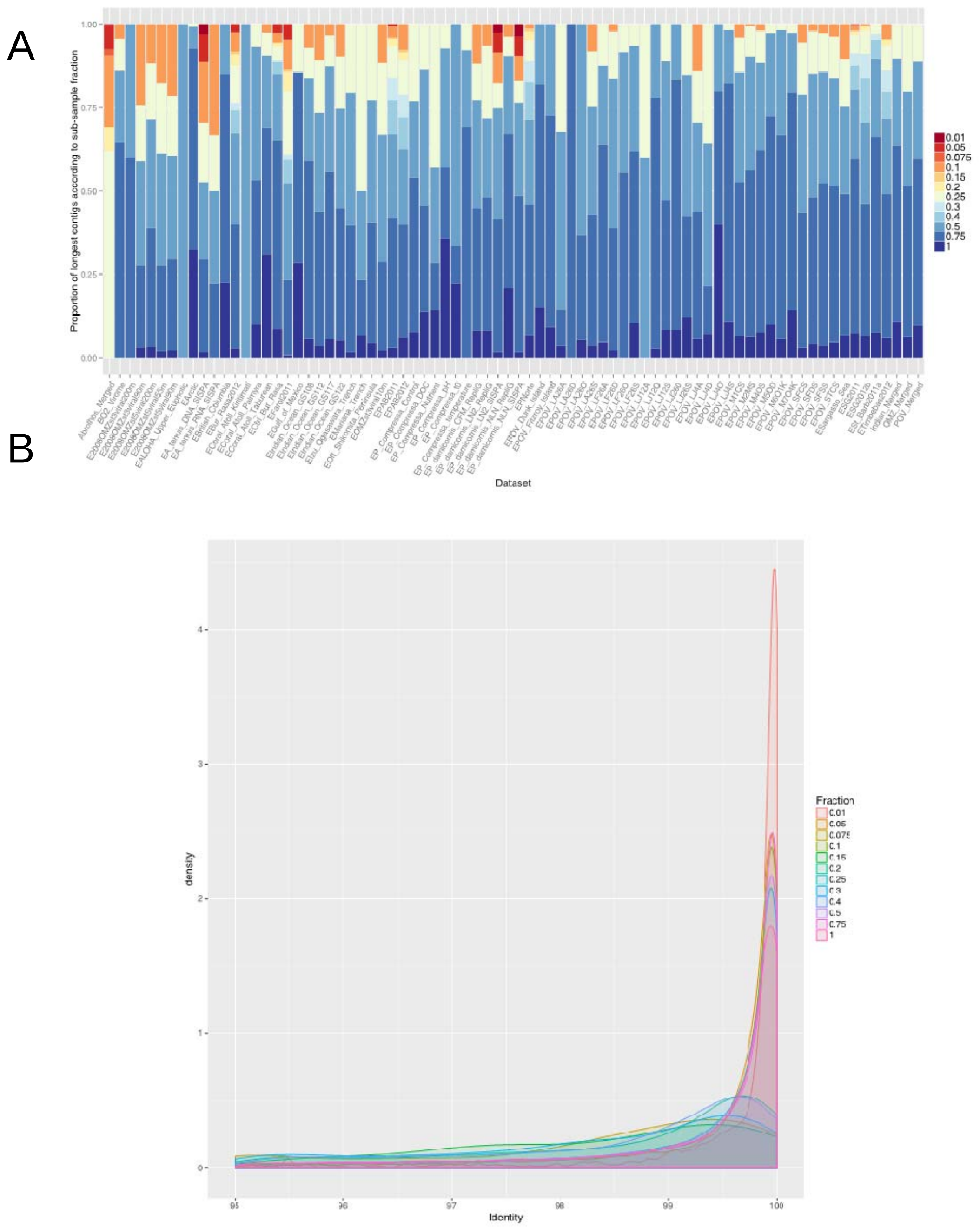


**Supplementary Figure 1:** Effect of random sub-sampling on virome assembly. Stacked bar plots displaying the proportion of contigs obtained for each sub-sample (color coded according to subsample size fractions) across each dataset. B) A density plot displaying the distribution of identity values of the contigs to the cluster representative according to the subsample fraction from which they are derived. The contigs from smaller subsamples displayed high identity (> 98%) to the cluster representative.

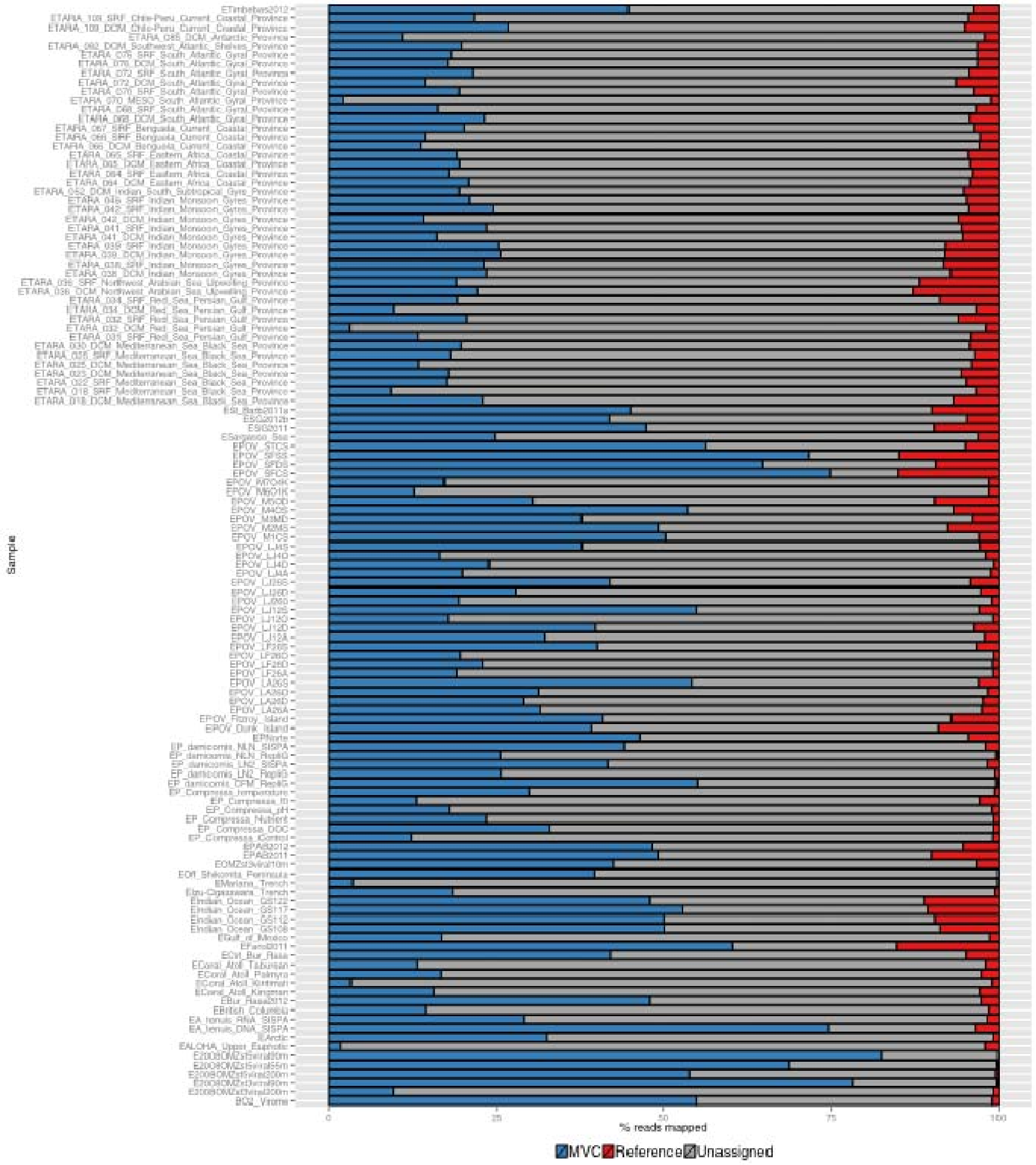


**Supplementary Figure 2:** Relative abundance of Unassigned reads (grey) or mapped to MVCs(blue) or Reference Phage Genomes (Red) in the 121 analysed marine viromes.

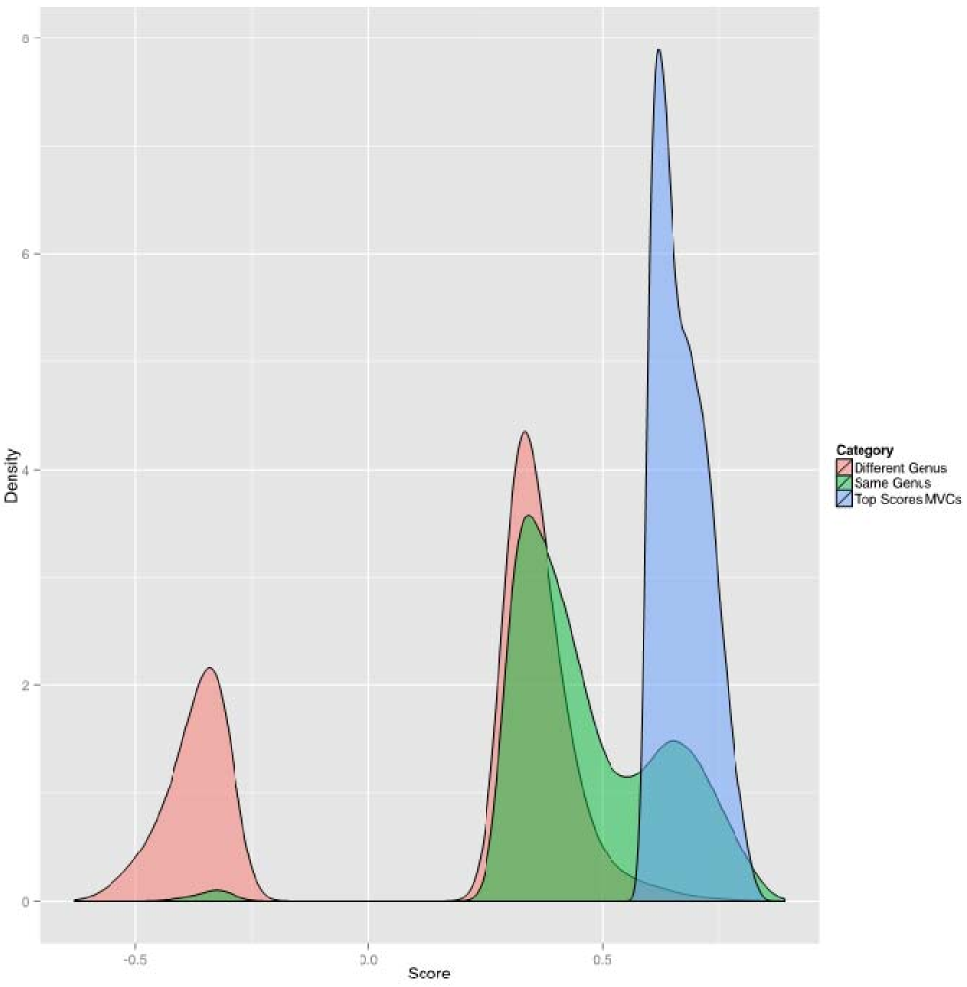


**Supplementary Figure 3:** Distribution of the SparCC correlation scores between prokaryotic viruses that infect a host of the same (green) or a different (red) genus. The top scores used to assign hosts to the MVCs are displayed in blue.

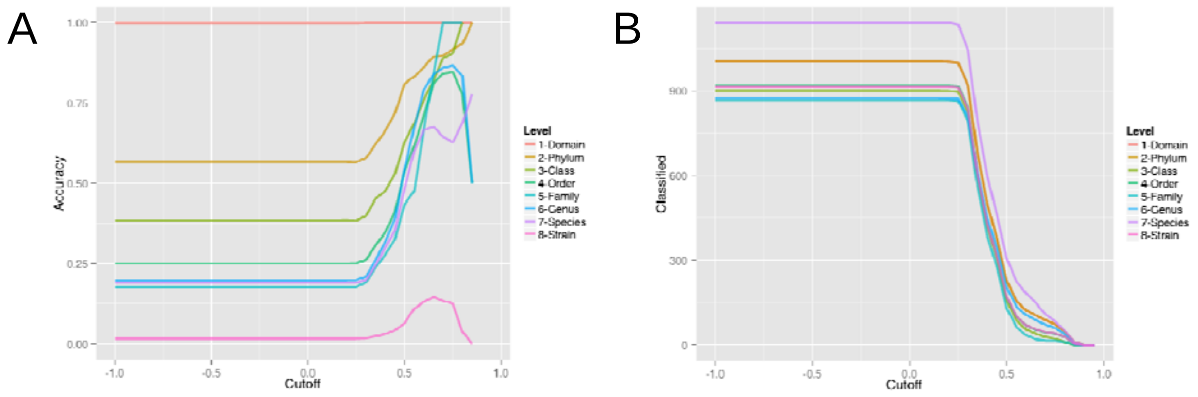


**Supplementary Figure 4:** Performance of the predictions based on the correlation scores for each taxonomic level. The X axis displays the minimum score of a correlation to be considered. A) The Y axis shows the accuracy of prediction. B) The Y axis shows the number of correlations in the network that fall within the established cut-off. The strongest scores detected (used for host prediction) were never below ~0.25. Hence, the lack of change in accuracy or the amount of predictions when the cut-off is set below the 0.25 threshold.

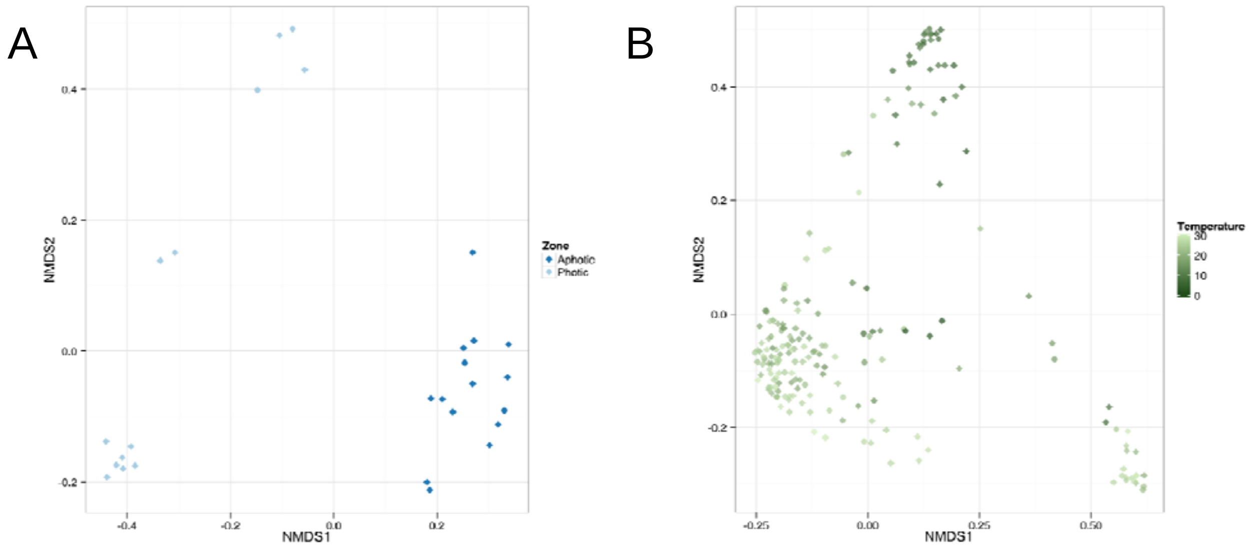


**Supplementary Figure 5:** Metagenome non-metric multidimensional scaling. The Manhattan distances were calculated based on prokaryote relative abundances estimated from cellular fraction microbial metagenomes and used as input for a NMDS analysis. The samples are colour coded according to their environmental features. A) SAO metagenomes. B) Tara oceans metagenomes.

**Supplementary Table 1:** Description of samples used to obtain the MVCs.

| File | Reads | Bases | Average_read_length | Reference |
|---|---|---|---|---|
| EArctic.fasta | 686209 | 68059812 | 99.1823365768 | (Angly et al., 2006) |
| EBritish_Columbia.fasta | 414964 | 42392364 | 102.1591366962 | (Angly et al., 2006) |
| EGulf_of_Mexico.fasta | 262501 | 26674941 | 101.6184357393 | (Angly et al., 2006) |
| ESargasso_Sea.fasta | 397939 | 41510756 | 104.3143697903 | (Angly et al., 2006) |
| E2008OMZst3viral200m.fasta | 96706 | 15866963 | 164.0742353112 | (Cassman et al., 2012) |
| E2008OMZst3viral90m.fasta | 361488 | 91589530 | 253.3681062719 | (Cassman et al., 2012) |
| E2008OMZst5viral200m.fasta | 163531 | 39736791 | 242.9924051097 | (Cassman et al., 2012) |
| E2008OMZst5viral55m.fasta | 226628 | 55711738 | 245.8290149496 | (Cassman et al., 2012) |
| E2008OMZst5viral90m.fasta | 300835 | 75835231 | 252.0824737813 | (Cassman et al., 2012) |
| EOMZst3viral10m.fasta | 128441 | 31647476 | 246.3969916148 | (Cassman et al., 2012) |
| ECoral_Atoll_Kingman.fasta | 93744 | 10159186 | 108.3715864482 | (Dinsdale et al., 2008) |
| ECoral_Atoll_Kiritimati.fasta | 279882 | 30957642 | 110.6096211975 | (Dinsdale et al., 2008) |
| ECoral_Atoll_Palmyra.fasta | 318178 | 33347087 | 104.8063882481 | (Dinsdale et al., 2008) |
| ECoral_Atoll_Taburean.fasta | 378475 | 39422223 | 104.1607054627 | (Dinsdale et al., 2008) |
| EPOV_Dunk_Island.fasta | 116855 | 28955563 | 247.7905352788 | (Hurwitz & Sullivan, 2013) |
| EPOV_Fitzroy_Island.fasta | 82739 | 20889623 | 252.4761358005 | (Hurwitz & Sullivan, 2013) |
| EPOV_LA26A.fasta | 42118 | 13594261 | 322.7660620162 | (Hurwitz & Sullivan, 2013) |
| EPOV_LA26D.fasta | 68516 | 18627549 | 271.8715190612 | (Hurwitz & Sullivan, 2013) |
| EPOV_LA26O.fasta | 70596 | 19933260 | 282.356790753 | (Hurwitz & Sullivan, 2013) |
| EPOV_LA26S.fasta | 165256 | 48389605 | 292.8160248342 | (Hurwitz & Sullivan, 2013) |
| EPOV_LF26A.fasta | 167616 | 48027477 | 286.5327713345 | (Hurwitz & Sullivan, 2013) |
| EPOV_LF26D.fasta | 125896 | 33423420 | 265.4843680498 | (Hurwitz & Sullivan, 2013) |
| EPOV_LF26O.fasta | 147537 | 40546266 | 274.8210008337 | (Hurwitz & Sullivan, 2013) |
| EPOV_LF26S.fasta | 192685 | 59738160 | 310.0301528401 | (Hurwitz & Sullivan, 2013) |
| EPOV_LJ12A.fasta | 58108 | 20543960 | 353.5478763681 | (Hurwitz & Sullivan, 2013) |
| EPOV_LJ12D.fasta | 49914 | 13188982 | 264.234122691 | (Hurwitz & Sullivan, 2013) |
| EPOV_LJ12O.fasta | 122565 | 45941366 | 374.83266838 | (Hurwitz & Sullivan, 2013) |
| EPOV_LJ12S.fasta | 92415 | 22582412 | 244.3587296435 | (Hurwitz & Sullivan, 2013) |
| EPOV_LJ260.fasta | 101179 | 28416547 | 280.8541989939 | (Hurwitz & Sullivan, 2013) |
| EPOV_LJ26D.fasta | 55332 | 14809960 | 267.6563290682 | (Hurwitz & Sullivan, 2013) |
| EPOV_LJ26S.fasta | 75036 | 19485257 | 259.6787808519 | (Hurwitz & Sullivan, 2013) |
| EPOV_LJ4A.fasta | 136876 | 44526523 | 325.305553932 | (Hurwitz & Sullivan, 2013) |
| EPOV_LJ4D.fasta | 98478 | 24169901 | 245.4345234469 | (Hurwitz & Sullivan, 2013) |
| EPOV_LJ4O.fasta | 97126 | 31475134 | 324.0649671561 | (Hurwitz & Sullivan, 2013) |
| EPOV_LJ4S.fasta | 107244 | 25738801 | 240.002247212 | (Hurwitz & Sullivan, 2013) |
| EPOV_M1CS.fasta | 303519 | 105200203 | 346.6017053298 | (Hurwitz & Sullivan, 2013) |
| EPOV_M2MS.fasta | 321754 | 92857788 | 288.5987058436 | (Hurwitz & Sullivan, 2013) |
| EPOV_M3MD.fasta | 31528 | 10927215 | 346.5876363867 | (Hurwitz & Sullivan, 2013) |
| EPOV_M4OS.fasta | 203238 | 52389322 | 257.7732609059 | (Hurwitz & Sullivan, 2013) |
| EPOV_M5OD.fasta | 156509 | 44009470 | 281.1944999968 | (Hurwitz & Sullivan, 2013) |
| EPOV_M6O1K.fasta | 225833 | 66311342 | 293.6299920738 | (Hurwitz & Sullivan, 2013) |
| EPOV_M7O4K.fasta | 144588 | 40451144 | 279.7683348549 | (Hurwitz & Sullivan, 2013) |
| EPOV_SFCS.fasta | 487339 | 191183563 | 392.3009711925 | (Hurwitz & Sullivan, 2013) |
| EPOV_SFDS.fasta | 645463 | 218687035 | 338.8064614083 | (Hurwitz & Sullivan, 2013) |
| EPOV_SFSS.fasta | 504826 | 173099948 | 342.8903186444 | (Hurwitz & Sullivan, 2013) |
| EPOV_STCS.fasta | 821404 | 246312846 | 299.8680868367 | (Hurwitz & Sullivan, 2013) |
| BO2_Virome.fasta | 2392879 | 855153008 | 357.3741121051 | (Solonenko et al., 2013) |
| EP_Compressa_Control.fasta | 39191 | 4065098 | 103.7252940726 | (Vega Thurber et al., 2008) |
| EP_Compressa_DOC.fasta | 35409 | 3620409 | 102.245446073 | (Vega Thurber et al., 2008) |
| EP_Compressa_Nutrient.fasta | 34139 | 3661593 | 107.2554263452 | (Vega Thurber et al., 2008) |
| EP_Compressa_pH.fasta | 49949 | 5234100 | 104.7888846624 | (Vega Thurber et al., 2008) |
| EP_Compressa_t0.fasta | 39113 | 3965136 | 101.3764221614 | (Vega Thurber et al., 2008) |
| EP_Compressa_temperature.fasta | 38482 | 4364749 | 113.4231328933 | (Vega Thurber et al., 2008) |
| EA_tenuis_DNA_SISPA.fasta | 1706540 | 528460555 | 309.6678396053 | (Weynberg et al., 2014) |
| EA_tenuis_RNA_SISPA.fasta | 734031 | 215417970 | 293.4725781336 | (Weynberg et al., 2014) |
| EP_damicornis_CFM_RepliG.fasta | 370135 | 100597355 | 271.785578235 | (Weynberg et al., 2014) |
| EP_damicornis_LN2_RepliG.fasta | 292476 | 82138284 | 280.8376892463 | (Weynberg et al., 2014) |
| EP_damicornis_LN2_SISPA.fasta | 2000000 | 585709573 | 292.8547865 | (Weynberg et al., 2014) |
| EP_damicornis_NLN_RepliG.fasta | 435494 | 122300721 | 280.8321607186 | (Weynberg et al., 2014) |
| EP_damicornis_NLN_SISPA.fasta | 2000000 | 604563712 | 302.281856 | (Weynberg et al., 2014) |
| EIndian_Ocean_GS108.fasta | 320104 | 125242428 | 391.2554294854 | (Williamson et al., 2012) |
| EIndian_Ocean_GS112.fasta | 494832 | 178668377 | 361.0687607107 | (Williamson et al., 2012) |
| EIndian_Ocean_GS117.fasta | 480375 | 194370437 | 404.6222992454 | (Williamson et al., 2012) |
| EIndian_Ocean_GS122.fasta | 341386 | 119422171 | 349.8156661375 | (Williamson et al., 2012) |
| EIzu-Ogasawara_Trench.fasta | 46458 | 15606815 | 335.9338542339 | (Yoshida et al., 2013) |
| EMariana_Trench.fasta | 49584 | 13814785 | 278.6137665376 | (Yoshida et al., 2013) |
| EOff_Shikomita_Peninsula.fasta | 76498 | 27935812 | 365.1835603545 | (Yoshida et al., 2013) |
| EBur_Rasa2012.fasta | 1943193 | 324123509 | 166.799442464 | Silveira et al., 2016. In press |
| ECtrl_Bur_Rasa.fasta | 2927179 | 565986037 | 193.3554582757 | Silveira et al., 2016. In press |
| EFarol2011.fasta | 5309048 | 1111783405 | 209.4129503067 | Silveira et al., 2016. In press |
| EPAB2011.fasta | 3353950 | 657983006 | 196.1815191043 | Silveira et al., 2016. In press |
| EPAB2012.fasta | 870827 | 182837271 | 209.9582018013 | Silveira et al., 2016. In press |

| Sample | Abrolhos_Season | POV_Depth | TARA_Temp |
|---|---|---|---|
| BO2_Virome | ignore | ignore | ignore |
| E2008OMZst3viral200m | ignore | ignore | ignore |
| E2008OMZst3viral90m | ignore | ignore | ignore |
| E2008OMZst5viral200m | ignore | ignore | ignore |
| E2008OMZst5viral55m | ignore | ignore | ignore |
| E2008OMZst5viral90m | ignore | ignore | ignore |
| EALOHA_Upper_Euphotic | ignore | ignore | ignore |
| EA_tenuis_DNA_SISPA | ignore | ignore | ignore |
| EA_tenuis_RNA_SISPA | ignore | ignore | ignore |
| EArctic | ignore | ignore | ignore |
| EBritish_Columbia | ignore | ignore | ignore |
| EBur_Rasa2012 | ignore | ignore | ignore |
| ECoral_Atoll_Kingman | ignore | ignore | ignore |
| ECoral_Atoll_Kiritimati | ignore | ignore | ignore |
| ECoral_Atoll_Palmyra | ignore | ignore | ignore |
| ECoral_Atoll_Taburean | ignore | ignore | ignore |
| ECtrl_Bur_Rasa | ignore | ignore | ignore |
| EFarol2011 | 2011 | ignore | ignore |
| EGulf_of_Mexico | ignore | ignore | ignore |
| EIndian_Ocean_GS108 | ignore | ignore | ignore |
| EIndian_Ocean_GS112 | ignore | ignore | ignore |
| EIndian_Ocean_GS117 | ignore | ignore | ignore |
| EIndian_Ocean_GS122 | ignore | ignore | ignore |
| EIzu-Ogasaw ara_Trench | ignore | ignore | ignore |
| EMariana_Trench | ignore | ignore | ignore |
| EOMZst3viral10m | ignore | ignore | ignore |
| EOff_Shikomita_Peninsula | ignore | ignore | ignore |
| EPAB2011 | 2011 | ignore | ignore |
| EPAB2012 | 2012 | ignore | ignore |
| EPNorte | 2012 | ignore | ignore |
| EPOV_Dunk_Island | ignore | Photic | ignore |
| EPOV_Fitzroy_Island | ignore | Photic | ignore |
| EPOV_LA26A | ignore | Aphotic | ignore |
| EPOV_LA26D | ignore | Aphotic | ignore |
| EPOV_LA26O | ignore | Aphotic | ignore |
| EPOV_LA26S | ignore | Photic | ignore |
| EPOV_LF26A | ignore | Aphotic | ignore |
| EPOV_LF26D | ignore | Aphotic | ignore |
| EPOV_LF26O | ignore | Aphotic | ignore |
| EPOV_LF26S | ignore | Photic | ignore |
| EPOV_LJ12A | ignore | Aphotic | ignore |
| EPOV_LJ12D | ignore | Aphotic | ignore |
| EPOV_LJ12O | ignore | Aphotic | ignore |
| EPOV_LJ12S | ignore | Photic | ignore |
| EPOV_LJ260 | ignore | Aphotic | ignore |
| EPOV_LJ26D | ignore | Aphotic | ignore |
| EPOV_LJ26S | ignore | Photic | ignore |
| EPOV_LJ4A | ignore | Aphotic | ignore |
| EPOV_LJ4D | ignore | Aphotic | ignore |
| EPOV_LJ4O | ignore | Aphotic | ignore |
| EPOV_LJ4S | ignore | Photic | ignore |
| EPOV_M1CS | ignore | Photic | ignore |
| EPOV_M2MS | ignore | Photic | ignore |
| EPOV_M3MD | ignore | Photic | ignore |
| EPOV_M4OS | ignore | Photic | ignore |
| EPOV_M5OD | ignore | Photic | ignore |
| EPOV_M6O1K | ignore | Aphotic | ignore |
| EPOV_M7O4K | ignore | Aphotic | ignore |
| EPOV_SFCS | ignore | Photic | ignore |
| EPOV_SFDS | ignore | Photic | ignore |
| EPOV_SFSS | ignore | Photic | ignore |
| EPOV_STCS | ignore | Photic | ignore |

Supplementary Table 2: Listing of sample groupings used for the enrichment analyzes.

| | | | |
|---|---|---|---|
| EP_Compressa_Control | ignore | ignore | ignore |
| EP_Compressa_DOC | ignore | ignore | ignore |
| EP_Compressa_Nutrient | ignore | ignore | ignore |
| EP_Compressa_pH | ignore | ignore | ignore |
| EP_Compressa_t0 | ignore | ignore | ignore |
| EP_Compressa_temperature | ignore | ignore | ignore |
| EP_damicornis_CFM_RepliG | ignore | ignore | ignore |
| EP_damicornis_LN2_RepliG | ignore | ignore | ignore |
| EP_damicornis_LN2_SISPA | ignore | ignore | ignore |
| EP_damicornis_NLN_RepliG | ignore | ignore | ignore |
| EP_damicornis_NLN_SISPA | ignore | ignore | ignore |
| ESG2011 | 2011 | ignore | ignore |
| ESG2012b | 2012 | ignore | ignore |
| ESargasso_Sea | ignore | ignore | ignore |
| ESt_Barb2011a | 2011 | ignore | ignore |
| ETARA_018_DCM_Mediterranean_Sea_Black_Sea_Province | ignore | ignore | Cold |
| ETARA_018_SRF_Mediterranean_Sea_Black_Sea_Province | ignore | ignore | Cold |
| ETARA_022_SRF_Mediterranean_Sea_Black_Sea_Province | ignore | ignore | Cold |
| ETARA_023_DCM_Mediterranean_Sea_Black_Sea_Province | ignore | ignore | Cold |
| ETARA_025_DCM_Mediterranean_Sea_Black_Sea_Province | ignore | ignore | Cold |
| ETARA_025_SRF_Mediterranean_Sea_Black_Sea_Province | ignore | ignore | Cold |
| ETARA_030_DCM_Mediterranean_Sea_Black_Sea_Province | ignore | ignore | Cold |
| ETARA_031_SRF_Red_Sea_Persian_Gulf_Province | ignore | ignore | Warm |
| ETARA_032_DCM_Red_Sea_Persian_Gulf_Province | ignore | ignore | Warm |
| ETARA_032_SRF_Red_Sea_Persian_Gulf_Province | ignore | ignore | Warm |
| ETARA_034_DCM_Red_Sea_Persian_Gulf_Province | ignore | ignore | Warm |
| ETARA_034_SRF_Red_Sea_Persian_Gulf_Province | ignore | ignore | Warm |
| ETARA_036_DCM_Northwest_Arabian_Sea_Upwelling_Province | ignore | ignore | Warm |
| ETARA_036_SRF_Northwest_Arabian_Sea_Upwelling_Province | ignore | ignore | Warm |
| ETARA_038_DCM_Indian_Monsoon_Gyres_Province | ignore | ignore | Warm |
| ETARA_038_SRF_Indian_Monsoon_Gyres_Province | ignore | ignore | Warm |
| ETARA_039_DCM_Indian_Monsoon_Gyres_Province | ignore | ignore | Warm |
| ETARA_039_SRF_Indian_Monsoon_Gyres_Province | ignore | ignore | Warm |
| ETARA_041_DCM_Indian_Monsoon_Gyres_Province | ignore | ignore | Warm |
| ETARA_041_SRF_Indian_Monsoon_Gyres_Province | ignore | ignore | Warm |
| ETARA_042_DCM_Indian_Monsoon_Gyres_Province | ignore | ignore | Warm |
| ETARA_042_SRF_Indian_Monsoon_Gyres_Province | ignore | ignore | Warm |
| ETARA_046_SRF_Indian_Monsoon_Gyres_Province | ignore | ignore | Warm |
| ETARA_052_DCM_Indian_South_Subtropical_Gyre_Province | ignore | ignore | Warm |
| ETARA_064_DCM_Eastern_Africa_Coastal_Province | ignore | ignore | Warm |
| ETARA_064_SRF_Eastern_Africa_Coastal_Province | ignore | ignore | Warm |
| ETARA_065_DCM_Eastern_Africa_Coastal_Province | ignore | ignore | Warm |
| ETARA_065_SRF_Eastern_Africa_Coastal_Province | ignore | ignore | Warm |
| ETARA_066_DCM_Benguela_Current_Coastal_Province | ignore | ignore | Cold |
| ETARA_066_SRF_Benguela_Current_Coastal_Province | ignore | ignore | Cold |
| ETARA_067_SRF_Benguela_Current_Coastal_Province | ignore | ignore | Cold |
| ETARA_068_DCM_South_Atlantic_Gyral_Province | ignore | ignore | Cold |
| ETARA_068_SRF_South_Atlantic_Gyral_Province | ignore | ignore | Cold |
| ETARA_070_MESO_South_Atlantic_Gyral_Province | ignore | ignore | Cold |
| ETARA_070_SRF_South_Atlantic_Gyral_Province | ignore | ignore | Cold |
| ETARA_072_DCM_South_Atlantic_Gyral_Province | ignore | ignore | Warm |
| ETARA_072_SRF_South_Atlantic_Gyral_Province | ignore | ignore | Warm |
| ETARA_076_DCM_South_Atlantic_Gyral_Province | ignore | ignore | Cold |
| ETARA_076_SRF_South_Atlantic_Gyral_Province | ignore | ignore | Cold |
| ETARA_082_DCM_Southwest_Atlantic_Shelves_Province | ignore | ignore | Cold |
| ETARA_085_DCM_Antarctic_Province | ignore | ignore | Cold |
| ETARA_109_DCM_Chile-Peru_Current_Coastal_Province | ignore | ignore | Warm |
| ETARA_109_SRF_Chile-Peru_Current_Coastal_Province | ignore | ignore | Warm |
| ETimbebas2012 | 2012 | ignore | ignore |

**Supplementary Table 3**: Correlation scores between the viruses and the hosts across the Tara Oceans samples calculated at the genus level.

| Microbe | Spearman_Score_Abundance | p_value_Abundance | Spearman_Score_VHR | p_value_VHR | Intercept | Coefficient | Median_VHR |
|---|---|---|---|---|---|---|---|
| Acholeplasma | -0.0897435897 | 0.6551148366 | -0.7918192918 | 2.77485041906018E-006 | 2.6390947056 | -221.356320944 | 0.0006731856 |
| Burkholderia | 0.0706766917 | 0.7673301273 | -0.7518796992 | 0.0002005705 | 0.3933706386 | -0.4911575602 | 0.0778090613 |
| Candidatus_Pelagibacter | 0.7207207207 | 1.91486869488262E-006 | -0.5933075933 | 0.0001825509 | 7.0776174966 | -0.206169233 | 0.5096964592 |
| Candidatus_Puniceispirillum | 0.0535714286 | 0.8525093768 | -0.7178571429 | 0.0035697913 | 0.8253670301 | -0.482693615 | 0.4417823791 |
| Clostridium | 0.1605616606 | 0.421987618 | -0.8424908425 | 1.63711885751082E-006 | 1.5244503504 | -2.4583227373 | 0.0886579336 |
| Croceibacter | 0.2295652174 | 0.2792184609 | -0.8695652174 | 2.51572197322635E-006 | 0.7746601981 | -30.9133058485 | 0.0011361179 |
| Exiguobacterium | -0.0457368718 | 0.8403574467 | -0.7312252964 | 0.0001678697 | 0.3507201017 | -4.4213806776 | 0.0005415427 |
| Fusobacterium | 0.3415384615 | 0.0882030026 | -0.8618803419 | 1.88156236780931E-006 | 0.1832144858 | -0.3048264016 | 0.1021796689 |
| Haloquadratum | -0.0336122076 | 0.8412189805 | -0.0697196446 | 0.6774607402 | 3.3308065431 | -13789.8936628182 | 0 |
| Leptospira | -0.0012870013 | 0.994621753 | -0.7292149292 | 1.41832304552279E-006 | 1.8757868748 | -10.1811706671 | 0.0117860692 |
| Leuconostoc | 0.3081554461 | 0.1107833117 | -0.741652983 | 1.15855424126558E-005 | 0.7661340972 | -21.4115177414 | 0.0034241717 |
| Magnetospirillum | -0.2631578947 | 0.2751840936 | -0.601754386 | 0.0075105622 | 0.3000664676 | -0.516229263 | 0.0062252617 |
| Marinomonas | -0.1168831169 | 0.6127360376 | -0.6662337662 | 0.0013002749 | 1.1968707761 | -599.2463695185 | 0.0003126992 |
| Methanobacterium | -0.0222305556 | 0.8976015663 | -0.2011528456 | 0.2394372755 | 1.3199809909 | -851.2545217336 | 2.33084931317066E-005 |
| Myxococcus | 0.0298584299 | 0.8626206951 | -0.502960103 | 0.0020146217 | 0.6104927701 | -19.9990135023 | 0.0007479578 |
| Porphyromonas | 0.0887507633 | 0.6945000219 | -0.2560770433 | 0.2500183316 | 0.289146152 | -2.8836058149 | 0.0009241011 |
| Prochlorococcus | 0.4062753036 | 0.0107797241 | 0.0253036437 | 0.8783670412 | 15.8161328076 | -2.2742458066 | 0.1961558333 |
| Rhodobacter | -0.5 | 0.1214291558 | -0.7818181818 | 0.0070121129 | 0.2041216285 | -0.091966454 | 0.0504024325 |
| Rhodopirellula | -0.0207715235 | 0.9250525748 | -0.5548964139 | 0.0059921124 | 0.790250522 | -96.4408425634 | 0.000504267 |
| Spiroplasma | 0.2393162393 | 0.2282881749 | -0.7490842491 | 1.24966777067635E-005 | 1.7682111785 | -29.3523367065 | 0.0064639359 |
| Staphylococcus | -0.0931451613 | 0.6169586777 | -0.9116935484 | 7.55137262061412E-008 | 1.238667744 | -2.5510849631 | 0.0835727133 |
| Streptococcus | -0.3128851541 | 0.0676570391 | -0.943977591 | 0 | 1.8591246303 | -11.8458256952 | 0.0416806827 |
| Sulfolobus | -0.4138904574 | 0.0566759494 | -0.9480519481 | 3.97738009413851E-006 | 1.2540135467 | -62.4437065058 | 0.0013793744 |
| Synechococcus | 0.4864957265 | 0.0126122509 | -0.4844444444 | 0.0130342351 | 2.4460221709 | -0.0702163975 | 4.9054168419 |
| Leptotrichia | -0.0285714286 | 1 | -0.9428571429 | 0.0166666667 | 0.6836752584 | -15.1989367014 | 0.0186075988 |
| Ruegeria | 0.103030303 | 0.785018104 | -0.4666666667 | 0.1782193293 | 0.2487522074 | -3.1218398561 | 0.0124057428 |
| Atopobium | -0.358554489 | 0.1018781887 | -0.8430265387 | 0.000000871 | 0.6696239452 | -4.4327296023 | 0.0028153489 |
| Bifidobacterium | 0.2 | 0.4100098244 | -0.601754386 | 0.0075105622 | 0.3162132879 | 0.1084235625 | 0.0109659178 |
| Lactobacillus | -0.0303030303 | 0.9457098084 | -0.9757575758 | 0 | 0.9724045537 | -1.4384026105 | 0.1180761148 |
| Mycoplasma | 0.3552419355 | 0.0505457136 | -0.8524193548 | 4.8258055809074E-007 | 0.9993676428 | -33.7842023949 | 0.003027669 |
| Prevotella | -0.1823529412 | 0.4979213725 | -0.9764705882 | 0 | 2.3123656689 | -64.3651237005 | 0.0024500631 |
| Shewanella | -0.1 | 0.95 | -0.9 | 0.0833333333 | 0.7163509017 | -0.9440151482 | 0.0276617615 |
| Spirochaeta | 0.0285714286 | 1 | -0.0857142857 | 0.9194444444 | 0.1415578274 | -13.1350742861 | 0.0013635406 |
| Corynebacterium | -0.0714285714 | 0.9063492063 | -0.3571428571 | 0.4444444444 | 0.3798208917 | 1.3208445271 | 0.0049118479 |
| Flavobacterium | 0.1281761717 | 0.568312186 | -0.8927159797 | 3.19706976253081E-006 | 1.1341277571 | -0.1239339403 | 0.6624901891 |
| Neisseria | -0.2142857143 | 0.6615079365 | -0.6428571429 | 0.1388888889 | 0.1934000597 | -0.6000173031 | 0.046770624 |
| Vibrio | -0.3696969697 | 0.2956040892 | -0.9757575758 | 0 | 1.0898577702 | -4.3502075622 | 0.0343918727 |
| Brachyspira | 0 | 1 | -0.75 | 0.0662698413 | 0.6816891301 | -26.8040952435 | 0.0062675438 |
| Coprococcus | -0.0286978514 | 0.9224205467 | -0.7483516645 | 0.0020787303 | 0.5972249469 | -128.5141800102 | 0.000224633 |
| Treponema | -0.6666666667 | 0.0830853175 | -0.9523809524 | 0.001140873 | 1.2788442978 | -393.2300271252 | 0.000489972 |
| Thermus | 0.6967032967 | 0.007343265 | -0.5912087912 | 0.0288937236 | 0.2070740535 | -1.7042496078 | 0.0525676916 |
| Haloferax | 0.1644325947 | 0.5581408249 | -0.2252011623 | 0.4196781971 | 0.4128272456 | -7.3942072781 | 0.0029201669 |
| Olsenella | -0.7181818182 | 0.0167992341 | -0.8 | 0.0052015025 | 0.3364084675 | -4.7355863648 | 0.0018903593 |
| Selenomonas | -0.1428571429 | 0.8027777778 | -0.7714285714 | 0.1027777778 | 1.0847197092 | -75.1076237115 | 0.0036546489 |
| Cellulophaga | 0.6 | 0.2416666667 | -0.9428571429 | 0.0166666667 | 1.6722300243 | -9.2681555217 | 0.0513465397 |
| Desulfovibrio | -0.2 | 0.7138888889 | -0.6 | 0.2416666667 | 0.1042627889 | -0.2308270493 | 0.064935013 |
| Bacillus | 0.6428571429 | 0.1388888889 | -0.8928571429 | 0.0123015873 | 0.8440946301 | -0.1930062559 | 0.2958520169 |
| Xylella | 0.6428571429 | 0.1388888889 | -0.8928571429 | 0.0123015873 | 1.0002495561 | -28.2940329388 | 0.0048490512 |

**Supplementary Table 4**: Correlation scores between the viruses and the hosts across the Tara Oceans samples calculated at the phylum level.

| Microbe | Spearman_Score_Abundance | p_value_Abundance | Spearman_Score_VHR | p_value_VHR | Intercept | Coefficient | Median_VHR |
|---|---|---|---|---|---|---|---|
| Bacteroidetes | 0.487854251 | 0.0018440462 | -0.0587044534 | 0.7218046355 | 6.5718123628 | -0.8691127829 | 0.2942114454 |
| Proteobacteria | 0.5700404858 | 0.0001969337 | 0.0714574899 | 0.6645328164 | 41.6510547139 | -2.8456391386 | 0.4728114824 |
| Chlamydiae | 0.224785273 | 0.4824316437 | -0.3408034784 | 0.2783436757 | 1.3054541352 | -1125.5415903991 | 0.0001312777 |
| Cyanobacteria | 0.4287449393 | 0.0068892257 | -0.1935222672 | 0.2369237852 | 21.7154380513 | -1.5670240917 | 1.631312893 |
| Nitrospirae | -0.1552539658 | 0.4393705456 | -0.2415941814 | 0.2247315758 | 0.692990787 | -757.9028606154 | 0 |
| Chloroflexi | 0.1402597403 | 0.5427637571 | -0.687012987 | 0.0008070563 | 0.4365735353 | -17.4971416845 | 0.0012303541 |
| Synergistetes | -0.5303759999 | 0.0932731105 | -0.8560454736 | 0.0007716582 | 0.4207727833 | -77.497439152 | 0.0006656232 |
| Firmicutes | 0.0495951417 | 0.7636823368 | -0.5718623482 | 0.0001862272 | 7.1450479718 | -10.3560829904 | 0.1007878894 |
| Planctomycetes | 0.0298719772 | 0.8585100099 | -0.644162381 | 2.0270257907871E-005 | 1.1593147267 | -20.0764366062 | 0.0053903494 |
| Euryarchaeota | -0.3380566802 | 0.03586063 | -0.6386639676 | 1.9170771177978E-005 | 11.0498598531 | -1047.2704950545 | 0.0007424886 |
| Spirochaetes | 0.2519969362 | 0.1267868461 | -0.6544479702 | 1.37698760641859E-005 | 2.3641307508 | -19.8223758252 | 0.0128895901 |
| Crenarchaeota | 0.0786738155 | 0.6376082776 | -0.7439544808 | 4.98082163215247E-007 | 2.1539224633 | -108.6354586489 | 0.0013896698 |
| Actinobacteria | -0.1083451873 | 0.5218849548 | -0.7598387862 | 4.2867457596182E-007 | 2.1527612906 | -1.161736751 | 0.2030214715 |
| Deinococcus-Thermus | 0.0512091038 | 0.7627878024 | -0.8477951636 | 1.14534805773338E-008 | 0.5870313379 | -4.3343088481 | 0.0229846302 |
| Fusobacteria | -0.0440967283 | 0.7950393182 | -0.9229492651 | 0 | 0.9894272934 | -2.9219067318 | 0.0236006339 |
| Tenericutes | -0.14048583 | 0.3922637652 | -0.8967611336 | 0 | 3.4383676516 | -11.5564472183 | 0.0044451534 |

**Supplementary References:**


Angly FE., Felts B., Breitbart M., Salamon P., Edwards RA., Carlson C., Chan AM., Haynes M., Kelley S., Liu H., Mahaffy JM., Mueller JE., Nulton J., Olson R., Parsons R., Rayhawk S., Suttle CA., Rohwer F. 2006. The marine viromes of four oceanic regions. PLoS Biol 4:e368.

Cassman N., Prieto-Davo A., Walsh K., Silva GG., Angly F., Akhter S., Barott K., Busch J., McDole T., Haggerty JM., Willner D., Alarcon G., Ulloa O., DeLong EF., Dutilh BE., Rohwer F., Dinsdale EA. 2012. Oxygen minimum zones harbour novel viral communities with low diversity. Environ Microbiol 14:3043–3065.

Dinsdale EA., Pantos O., Smriga S., Edwards RA., Angly F., Hatay M., Hall D., Brown E., Haynes M., Krause L., Sala E., Stuart A., Thurber RV., Willis BL., Azam F., Knowlton N., Rohwer F. 2008. Microbial Ecology of Four Coral Atolls in the Northern Line Islands. 3.

Hurwitz BL., Sullivan MB. 2013. The Pacific Ocean virome (POV): a marine viral metagenomic dataset and associated protein clusters for quantitative viral ecology. PLoS

One 8:e57355.

Solonenko S a., Ignacio-Espinoza JC., Alberti A., Cruaud C., Hallam S., Konstantinidis K., Tyson G., Wincker P., Sullivan MB. 2013. Sequencing platform and library preparation choices impact viral metagenomes. BMC genomics 14:320.

Vega Thurber RL., Barott KL., Hall D., Liu H., Rodriguez-Mueller B., Desnues C., Edwards RA., Haynes M., Angly FE., Wegley L., Rohwer FL. 2008. Metagenomic analysis indicates that stressors induce production of herpes-like viruses in the coral Porites compressa. Proceedings of the National Academy of Sciences of the United States of America 105:18413–18418.

Weynberg KD., Wood-Charlson EM., Suttle CA., van Oppen MJ. 2014. Generating viral metagenomes from the coral holobiont. Front Microbiol 5:206.

Williamson SJ., Allen LZ., Lorenzi H a., Fadrosh DW., Brami D., Thiagarajan M., McCrow JP., Tovchigrechko A., Yooseph S., Venter JC. 2012. Metagenomic exploration of viruses throughout the Indian Ocean. PloS one 7:e42047.

Yoshida M., Takaki Y., Eitoku M., Nunoura T., Takai K. 2013. Metagenomic Analysis of Viral Communities in (Hado)Pelagic Sediments. PLoS ONE 8.
